# The Tobacco Habit

**Published:** 1888-02

**Authors:** 


### THE TOBACCO HABIT.

It is a fact which cannot be disputed, that boys who are persistent cigarette smokers do not reach perfect maturity. Their growth, both physically and intellectually, is retarded. Their nervous system is but imperfectly developed; digestion, sight and other important functions are seriously impaired. Irritability of the heart is one common consequence of the use of tobacco in any form in early life. Let all boys who use tobacco understand this; they can never hope to become MEN. They will grow old, and prematurely old, but true, manly development and vigor they can never attain; and for their chances of success as students and scholars, even the mild use of tobacco impairs them, and the persistent use wholly destroys them. Never, before the age of twenty-one is reached, should tobacco be indulged in, and its use might more wisely be delayed until the body has become fully and completely developed. Parents should see to it, and, if necessary, laws should be enacted, that this rule be strictly enforced. There is an awful responsibility here which all should feel, and do their utmost to stay the degeneration of our youth, which is threatened by this, one of the greatest curses known to us—the tobacco habit in boys.—*Boston Journal of Health.*

